# Supplementary material for: Mode of delivery modulates the intestinal microbiota and impacts the response to vaccination
Source: Nat Commun. 2022 Nov 15;13:6638. doi: 10.1038/s41467-022-34155-2 (PMC9666625; doi:10.1038/s41467-022-34155-2)
Supplement: Supplementary file 1 — Supplementary Information [file 41467_2022_34155_MOESM1_ESM.pdf]

Supplementary Material to:

Mode of delivery modulates the intestinal microbiota and impacts the response to vaccination

Authors: Emma M. de Koff, Debbie van Baarle, Marlies A. van Houten, Marta Reyman, Guy A.M. Berbers, Femke van den Ham, Mei Ling J.N. Chu, Elisabeth A.M. Sanders, Debby Bogaert, Susana Fuentes

Supplementary Figures

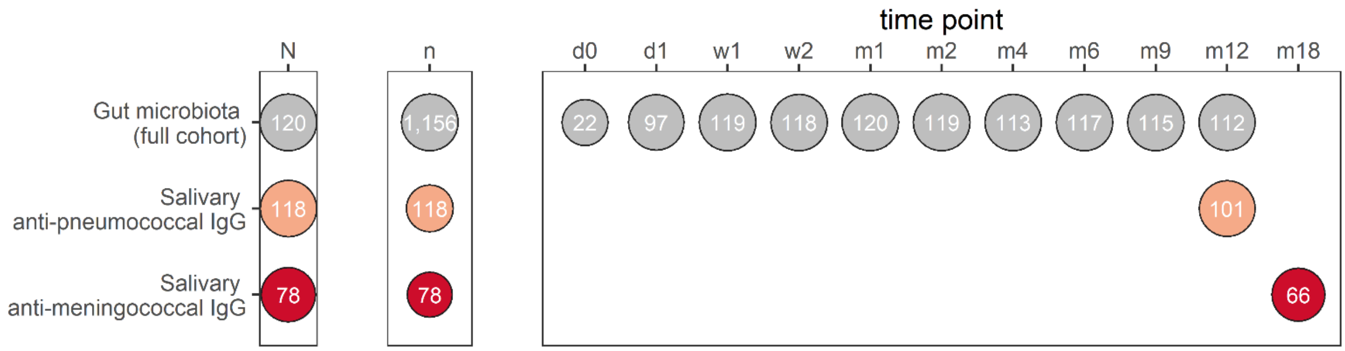

Supplementary Figure 1. Sample overview
Fecal samples were collected for gut microbiota characterization from 120 healthy infants at days (d)0 and 1, weeks (w)1 and 2, and months (m)1, 2, 4, 6, 9 and 12. Fecal samples were excluded from the analysis if they had insufficient bacterial DNA available (*n*=104). Saliva was collected from 118 infants at the age of 12 months for measuring anti-pneumococcal immunoglobulin G (IgG) and from 78 infants at the age of 18 months for measuring anti-meningococcal IgG. Saliva samples were excluded if infants did not receive their vaccinations in time (*n*=8 at month 12, *n*=1 at month 18), or if the saliva sample did not have a sufficient volume for laboratory analysis (n=8 at month 12, n=11 at month 18). N=total number of infants from which samples were collected; n=total number of available samples. Source data are provided in the Source Data file.

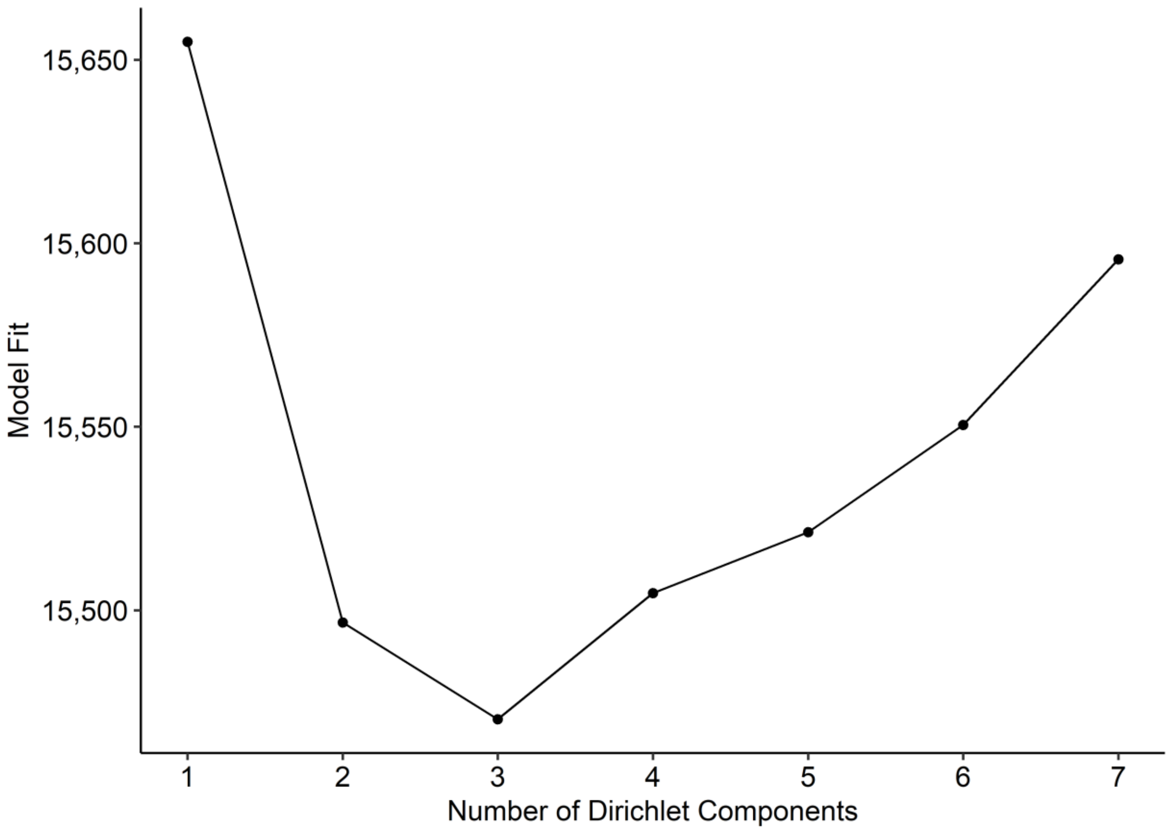

Supplementary Figure 2. Dirichlet multinomial mixture model fit.
Dirichlet multinomial mixture model identified 3 compositionally distinct community state types (CST) as the best model fit at the week 1 timepoint. Model fit was based on the Laplace approximation to the negative log model where a lower value indicates a better model fit. Source data are provided in the Source Data file.

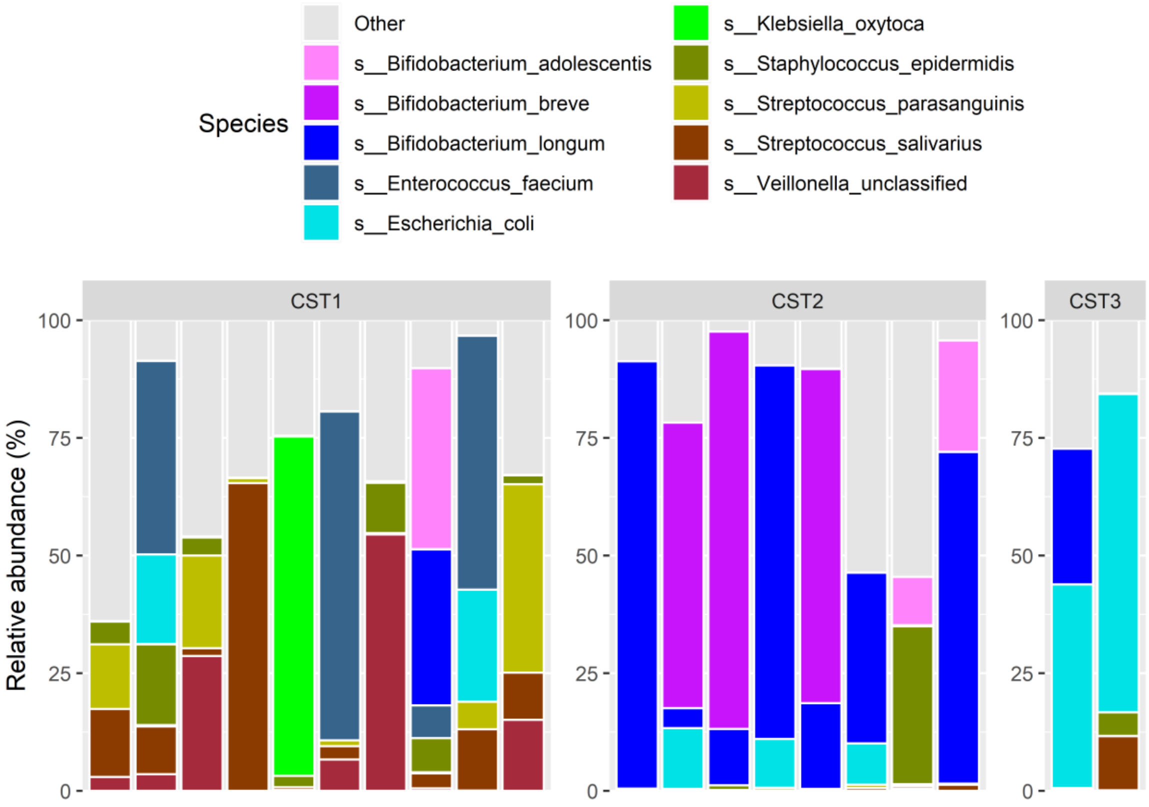

Supplementary Figure 3. Species-level composition of community state types at week 1 of age. Relative abundances of the top 10 species in 20 week 1 samples, determined by whole genome shotgun sequencing. Samples were ordered by week 1 community state type (CST). Source data are provided in the Source Data file.

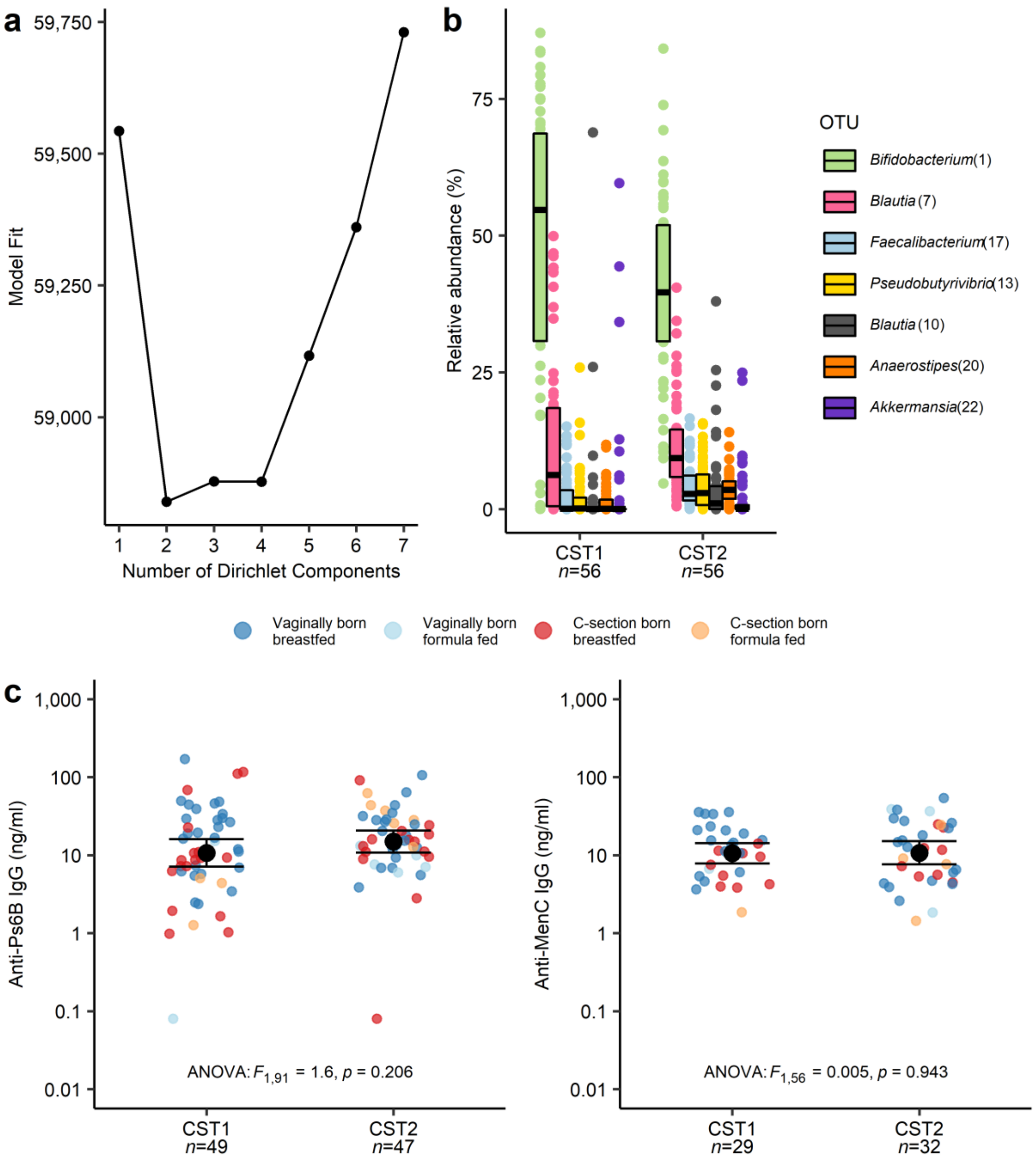

Supplementary Figure 4. Community state types at 12 months of age.
(A) Dirichlet multinomial mixture model identified two compositionally distinct community state types (CST) as the best model fit at the month 12 timepoint. Model fit was based on the Laplace approximation to the negative log model where a lower value indicates a better model fit. (B) Boxplot of relative abundances of the top 7 operational taxonomic units (OTUs) per community state type (CST) defined at 12 months of age. Boxes show medians with interquartile ranges. (C) Month 12 CSTs are plotted against anti-Ps6B IgG concentrations (left) and anti-MenC IgG concentrations (right). Dots are colored according to mode of delivery and feeding type from birth. Black dots and error bars represent geometric mean concentrations with 95% confidence intervals. Significance was assessed using two-sided ANOVA on log-transformed IgG concentrations, correcting for time between vaccination and IgG measurements. Source data are provided in the Source Data file.

Supplementary Tables

	Formula feeding vs breastfeeding		Vaginal birth vs C-section birth		Female vs male		AB vs no AB in first 3 months		Pets vs no pets in household		Formula feeding * vaginal birth	
Ps	*β* (95% CI)	*p*-value	*β* (95% CI)	*p*-value	*β* (95% CI)	*p*-value	*β* (95% CI)	*p*-value	*β* (95% CI)	*p*-value	*β* (95% CI)	*p*-value
1	0.57 (-0.19-1.33)	0.142	0.48 (0.01-0.96)	0.046	-0.09 (-0.52-0.34)	0.682	-0.26 (-0.91-0.39)	0.429	-0.19 (-0.62-0.24)	0.384	-1.87 (-3.00- -0.73)	0.002
4	-0.17 (-0.82-0.47)	0.596	0.26 (-0.14-0.66)	0.194	-0.01 (-0.37-0.36)	0.971	-0.29 (-0.83-0.26)	0.302	-0.38 (-0.74- -0.02)	0.041	-0.80 (-1.76-0.16)	0.100
5	0.35 (-0.27-0.97)	0.263	0.49 (0.11-0.87)	0.013	0.06 (-0.29-0.40)	0.751	-0.25 (-0.78-0.27)	0.340	-0.15 (-0.50-0.20)	0.394	-1.37 (-2.28- -0.45)	0.004
7F	0.35 (-0.29-1.00)	0.281	0.41 (0.01-0.81)	0.045	0.38 (0.02-0.75)	0.040	-0.32 (-0.87-0.23)	0.250	-0.25 (-0.62-0.11)	0.171	-1.46 (-2.42- -0.50)	0.003
9V	0.36 (-0.26-0.99)	0.246	0.36 (-0.02-0.75)	0.065	0.03 (-0.32-0.38)	0.874	-0.40 (-0.92-0.13)	0.140	-0.19 (-0.54-0.16)	0.291	-1.23 (-2.16- -0.31)	0.009
14	0.41 (-0.31-1.12)	0.262	0.32 (-0.12-0.76)	0.154	0.16 (-0.24-0.57)	0.422	-0.23 (-0.84-0.37)	0.444	-0.37 (-0.77-0.03)	0.070	-1.50 (-2.56- -0.44)	0.006
18C	-0.49 (-1.23-0.25)	0.191	0.05 (-0.40-0.51)	0.820	0.09 (-0.33-0.50)	0.674	-0.72 (-1.34--0.09)	0.025	-0.52 (-0.94--0.11)	0.014	-0.28 (-1.38-0.81)	0.610
19F	0.27 (-0.44-0.99)	0.452	0.29 (-0.16-0.73)	0.203	0.16 (-0.24-0.56)	0.434	-0.53 (-1.14-0.08)	0.087	-0.29 (-0.69-0.11)	0.154	-1.32 (-2.38- -0.26)	0.015
23F	0.10 (-0.60-0.80)	0.778	0.39 (-0.04-0.83)	0.076	-0.01 (-0.40-0.39)	0.978	-0.40 (-0.99-0.20)	0.187	-0.26 (-0.66-0.13)	0.186	-0.79 (-1.83-0.25)	0.135

Supplementary Table 1. Validation of associations between early-life characteristics and anti-pneumococcal IgG concentrations following vaccination.
Model coefficients (β) with 95% CI and p-values were computed with two-sided multivariable linear regression on log-transformed anti-pneumococcal concentrations per serotype (Ps; n=101). All analyses were corrected for time between vaccination and IgG measurement, but not for multiple comparisons.
Abbreviations: C-section = caesarean section; AB = antibiotics.

Ps	time interval	*β* (95% CI)	adjusted *p*-value
1	d1-w1	1.20 (-0.03 - 2.42)	0.065
	w1-w2	0.86 (-0.05 - 1.78)	0.065
4	d1-w1	1.07 (0.08 - 2.06)	0.055
	w1-w2	0.75 (-0.02 - 1.51)	0.055
5	d1-w1	1.00 (0.05 - 1.95)	0.040
	w1-w2	0.80 (0.09 - 1.51)	0.040
7F	d1-w1	0.72 (-0.29 - 1.73)	0.162
	w1-w2	0.96 (0.22 - 1.70)	0.024
9V	d1-w1	1.22 (0.29 - 2.15)	0.011
	w1-w2	1.02 (0.33 - 1.72)	0.009
14	d1-w1	1.25 (0.18 - 2.33)	0.023
	w1-w2	1.25 (0.43 - 2.08)	0.006
18C	d1-w1	1.77 (0.72 - 2.82)	0.002
	w1-w2	0.77 (-0.10 - 1.65)	0.083
19F	d1-w1	1.14 (0.13 - 2.15)	0.056
	w1-w2	0.41 (-0.38 - 1.20)	0.307
23F	d1-w1	1.01 (-0.11 - 2.13)	0.077
	w1-w2	1.15 (0.27 - 2.03)	0.023

Supplementary Table 2. Validation of associations between Bray-Curtis similarity and anti-pneumococcal IgG levels.

Model coefficients (β) with 95% CI and adjusted p-values were computed with two-sided multivariable linear regression on log-transformed anti-pneumococcal concentrations per serotype (Ps; n=101). All analyses were corrected for time between vaccination and IgG measurement and for multiple comparisons.

Abbreviations: Ps = pneumococcal serotype; d1=day 1; w1=week 1; w2=week 2.

	ANOVA		Post-hoc Tukey-Kramer tests					
	All CSTs		CST 1 vs. CST 2		CST 1 vs. CST3		CST 2 vs. CST 3	
Ps	*F* (2,95)	*p*-value	difference	adjusted *p*-value	difference	adjusted *p*-value	difference	adjusted *p*-value
1	3.2	0.043	-0.554	0.239	-0.765	0.068	-0.211	0.808
4	2.9	0.062	-0.450	0.267	-0.595	0.102	-0.145	0.869
5	3.6	0.031	-0.537	0.114	-0.469	0.188	0.067	0.965
7F	2.5	0.084	-0.481	0.203	-0.382	0.361	0.098	0.934
9V	3.1	0.049	-0.468	0.188	-0.512	0.135	-0.045	0.985
14	3.0	0.056	-0.540	0.204	-0.596	0.145	-0.056	0.982
18C	3.1	0.050	-0.580	0.179	-0.598	0.161	-0.018	0.998
19F	1.0	0.386	-0.297	0.576	-0.300	0.571	-0.002	1.000
23F	3.0	0.056	-0.642	0.139	-0.356	0.538	0.286	0.670

Supplementary Table 3. Validation of association between week 1 CST and IgG concentrations against pneumococcal serotypes.
All analyses were two-sided and corrected for time between vaccination and IgG measurement. Abbreviations: CST = community state type; Ps = pneumococcal serotype.

	Anti-Ps6B IgG				Anti-MenC IgG			
	Model without CST		Model with CST		Model without CST		Model with CST	
	β (95% CI)	*p*-value	*β* (95% CI)	*p*-value	*β* (95% CI)	*p*-value	*β* (95% CI)	*p*-value
(Intercept)	2.13 (1.71-2.55)	<0.0001	2.05 (1.62-2.48)	<0.0001	2.03 (1.71-2.35)	<0.0001	1.93 (1.59-2.27)	<0.0001
Vaginal birth	0.53 (-0.02-1.08)	0.060	0.04 (-0.74-0.83)	0.911	0.52 (0.12-0.91)	0.012	0.68 (0.14-1.22)	0.015
CST2 vs 1	*NA*	*NA*	0.72 (-0.12-1.57)	0.091	*NA*	*NA*	-0.14 (-0.69-0.40)	0.602
CST3 vs 1	*NA*	*NA*	0.60 (-0.25-1.45)	0.165	*NA*	*NA*	0.48 (-0.11-1.06)	0.107

Supplementary Table 4. CST as a mediator between mode of birth and anti-Ps6B and anti-MenC IgG concentrations.
Model coefficients (β) with 95% CI and adjusted p-values were computed with two-sided multivariable linear regression on log-transformed anti-Ps6B (n=101) and anti-MenC concentrations. All analyses were corrected for time between vaccination and IgG measurement.
Abbreviations: CST = community state type; NA = not applicable.

OTU	Interval number	Interval start	Interval end	Area	Association	*p*-value	Adjusted *p*-value	Validated for Ps*:
Bifidobacterium (1)	interval:1	0	5	7.2	Above median	0.013	0.027	1, 4, 5, 7F, 9V, 19F, 23F
Escherichia coli (2)	interval:1	0	41	82.0	Above median	0.003	0.013	1, 4, 5, 7F, 9V, 18C, 23F
Ruminococcus gnavus (9)	interval:1	0	16	20.5	Above median	0.016	0.031	7F
Pseudobutyrivibrio (13)	interval:1	0	20	-14.3	Below median	0.050	0.071	1, 4, 5, 7F, 9V, 23F
Anaerostipes (20)	interval:1	0	17	-8.6	Below median	0.064	0.084	5, 7F, 9V
Clostridium sensu stricto 1 (21)	interval:1	0	31	-46.0	Below median	0.023	0.040	5, 7F, 23F
Prevotella (25)	interval:1	0	23	-22.4	Below median	0.001	0.010	1, 4, 5, 7F, 9V, 14, 18C, 19F, 23F
Streptococcus pyogenes (26)	interval:1	0	33	-21.4	Below median	0.013	0.027	1, 9V, 14
Dorea (32)	interval:1	0	4	-5.2	Below median	0.070	0.089	4, 5, 7F, 9V
Bacteroides (53)	interval:1	0	48	42.7	Above median	0.002	0.010	1, 4, 5, 7F, 9V, 19F, 23F
Streptococcus (55)	interval:1	0	43	-50.3	Below median	0.024	0.040	1, 4, 9V
Lactococcus lactis (80)	interval:1	0	33	-35.7	Below median	0.001	0.010	1, 5, 7F, 9V, 14, 18C, 19F, 23F
Bifidobacterium (147)	interval:1	0	19	18.0	Above median	0.002	0.010	1, 4, 5, 7F, 19F, 23F
Escherichia/Shigella (185)	interval:1	0	36	46.7	Above median	0.002	0.010	1, 7F, 18C
Pseudomonas fluorescens (236)	interval:1	0	5	-3.7	Below median	0.088	0.099	5, 7F, 9V
Bacillales (255)	interval:1	0	39	-38.7	Below median	0.001	0.010	4, 5, 7F, 9V, 19F, 23F
Enterococcus (256)	interval:1	0	45	-48.8	Below median	0.002	0.010	1, 4, 5, 7F, 9V, 18C, 19F, 23F
Enterobacteriaceae (345)	interval:1	0	15	7.4	Above median	0.020	0.038	
Staphylococcaceae (382)	interval:1	0	58	-52.1	Below median	0.001	0.010	4, 5, 7F, 9V, 19F, 23F
Streptococcus gallolyticus (18)	interval:1	2	44	45.0	Above median	0.013	0.027	14
Bacteroides (433)	interval:1	3	8	2.0	Above median	0.023	0.040	4, 9V, 19F, 23F
Streptococcus (502)	interval:1	3	49	-42.3	Below median	0.002	0.010	1, 5, 7F, 9V, 23F
Bacteroides (19)	interval:1	4	47	58.0	Above median	0.021	0.039	1, 4, 5, 7F, 9V, 18C, 19F, 23F
Streptococcus (189)	interval:1	4	26	-26.4	Below median	0.007	0.019	1, 4, 5, 9V
Gardnerella (333)	interval:1	11	60	-51.8	Below median	0.004	0.014	1, 5, 9V, 14, 18C
Veillonella (366)	interval:1	11	62	-44.3	Below median	0.004	0.014	1, 5, 7F, 9V, 14, 23F
Bacteroides (35)	interval:1	12	47	42.3	Above median	0.009	0.022	1, 4, 5, 18C, 19F, 23F
Bilophila wadsworthia (136)	interval:1	14	70	52.7	Above median	0.006	0.018	1, 4, 5, 9V, 14, 23F

Subdoligranulum (38)	interval:2	15	30	13.4	Above median	0.006	0.018	1, 4, 14
Bifidobacterium bifidum (11)	interval:1	16	70	-101.4	Below median	0.026	0.041	9V, 14, 18C, 19F
Rothia (113)	interval:1	17	29	-9.1	Below median	0.062	0.084	4, 9V, 23F
Peptostreptococcus (168)	interval:1	17	70	50.8	Above median	0.081	0.095	9V, 23F
Bacteroides (249)	interval:1	17	70	39.4	Above median	0.002	0.010	4, 5, 7F, 9V, 19F, 23F
Streptococcus (69)	interval:1	18	55	-35.0	Below median	0.074	0.090	
Lactobacillus fermentum (75)	interval:1	18	30	-6.0	Below median	0.074	0.090	4
Veillonella (179)	interval:1	19	32	-7.3	Below median	0.029	0.045	1, 4, 5, 7F, 9V, 19F
Enterococcus faecium (5)	interval:1	20	23	-3.8	Below median	0.046	0.067	5, 7F, 23F
Bifidobacteriaceae (309)	interval:1	21	48	-14.5	Below median	0.089	0.099	4, 7F, 9V, 18C, 23F
Bifidobacterium animalis (41)	interval:1	22	64	-54.7	Below median	0.015	0.031	1, 5, 9V, 14, 18C, 19F, 23F
Bacteroides (48)	interval:1	24	41	14.6	Above median	0.065	0.084	14, 23F
Bacteroides (12)	interval:1	26	70	81.1	Above median	0.007	0.019	7F, 23F
Bifidobacterium (229)	interval:1	29	70	-40.3	Below median	0.005	0.017	9V, 14, 18C, 19F
Blautia (10)	interval:1	33	59	140.0	Above median	0.003	0.013	1, 4, 19F, 23F
Enterobacteriaceae (242)	interval:1	33	70	-42.4	Below median	0.002	0.010	9V, 18C, 19F, 23F
Klebsiella (4)	interval:1	34	70	-80.7	Below median	0.004	0.014	
Carnobacteriaceae (311)	interval:1	38	70	22.6	Above median	0.061	0.084	14, 19F
Bifidobacterium animalis (175)	interval:1	39	70	-27.0	Below median	0.082	0.095	14
Citrobacter sedlakii (288)	interval:1	46	70	-23.1	Below median	0.026	0.041	9V
Finegoldia (203)	interval:1	50	70	-20.0	Below median	0.034	0.051	
Blautia (7)	interval:1	57	70	-23.5	Below median	0.012	0.027	4, 5, 7F, 9V, 18C
Corynebacterium propinquum (79)	interval:1	66	70	-11.6	Below median	0.009	0.022	

Supplementary Table 5. Differentially abundant OTUs in the first 2 months of life between infants with above vs. below median anti-Ps6B IgG levels

Differential abundance analysis was performed using two-sided smoothing spline ANOVA (ss-ANOVA). All analyses were corrected for time between vaccination and IgG measurements and for multiple comparisons.

** Validation for other pneumococcal vaccine serotypes 1, 4, 5, 7F, 9V, 14, 18C, 19F and 23F was performed using the same method. OTUs were considered validated for a given serotype if they were significantly differentially abundant (adjusted p<0.100) and were associated with the same response category (above or below median).*

Abbreviations: Ps = pneumococcal serotype

OTU	Interval number	Interval start	Interval end	Area	Association	*p*-value	Adjusted *p*-value
Escherichia coli (2)	interval:1	0	13	23.2	Above median	0.062	0.072
Veillonella (8)	interval:1	0	7	-9.2	Below median	0.037	0.055
Peptostreptococcaceae (46)	interval:1	0	7	9.4	Above median	0.021	0.054
Bacteroides (65)	interval:1	0	26	25.8	Above median	0.014	0.054
Streptococcus (69)	interval:1	0	14	-18.5	Below median	0.023	0.054
Rothia (113)	interval:1	0	43	-52.8	Below median	0.027	0.054
Bifidobacteriaceae (299)	interval:1	0	16	-7.9	Below median	0.005	0.036
Lachnospiraceae (96)	interval:1	1	51	47.4	Above median	0.003	0.029
Collinsella (16)	interval:1	3	20	23.7	Above median	0.037	0.055
Veillonella (85)	interval:1	3	16	-20.9	Below median	0.002	0.029
Klebsiella (252)	interval:1	4	12	-10.8	Below median	0.001	0.029
Veillonella (368)	interval:1	5	14	-7.7	Below median	0.013	0.054
Bifidobacterium (218)	interval:1	6	42	-25.1	Below median	0.025	0.054
Clostridium sensu stricto 1 (24)	interval:1	7	20	-19.7	Below median	0.017	0.054
Escherichia Shigella (185)	interval:1	10	24	12.3	Above median	0.043	0.059
Bifidobacteriaceae (309)	interval:1	11	53	34.6	Above median	0.060	0.072
Enterococcaceae (251)	interval:1	13	39	-28.1	Below median	0.012	0.054
Veillonella (160)	interval:1	15	41	-29.0	Below median	0.034	0.055
Lactobacillus (49)	interval:1	21	55	-47.0	Below median	0.062	0.072
Bilophila wadsworthia (136)	interval:1	21	69	-54.8	Below median	0.022	0.054
bacterium NLAE zl C558 (45)	interval:1	26	69	54.9	Above median	0.068	0.076
Lactobacillus fermentum (75)	interval:1	33	69	-49.0	Below median	0.037	0.055
Lachnospiraceae (30)	interval:1	35	69	-39.3	Below median	0.023	0.054
Bifidobacterium breve (261)	interval:1	38	69	30.6	Above median	0.072	0.077
Streptococcus salivarius (6)	interval:1	40	69	-42.4	Below median	0.028	0.054
Enterobacteriaceae (281)	interval:1	45	62	-11.9	Below median	0.054	0.071
Clostridium butyricum (33)	interval:1	62	69	26.2	Above median	0.038	0.055

Supplementary table 6. Differentially abundant OTUs in the first 2 months of life between infants with above vs. below median anti-MenC IgG levels

Differential abundance analysis was performed using two-sided smoothing spline ANOVA (ss-ANOVA). All analyses were corrected for time between vaccination and IgG measurements and for multiple comparisons.

OTU	Interval number	Interval start	Interval end	Area	Association	*p*-value	Adjusted *p*-value
Lachnospira (89)	interval:1	32	169	60.8	Above median	0.013	0.052
Lachnospiraceae (30)	interval:1	82	134	-49.9	Below median	0.013	0.052
Pseudobutyrivibrio (132)	interval:1	90	381	144.2	Above median	0.030	0.066
Blautia (67)	interval:1	100	381	313.6	Above median	0.020	0.053
Fusicatenibacter saccharivorans (15)	interval:1	101	381	439.7	Above median	0.050	0.080
Pseudobutyrivibrio (13)	interval:1	125	381	353.8	Above median	0.004	0.036
Lachnospiraceae (306)	interval:1	130	381	130.4	Above median	0.023	0.054
Roseburia (77)	interval:1	155	381	284.2	Above median	0.019	0.053
Dorea (54)	interval:1	156	381	185.8	Above median	0.075	0.094
Blautia (28)	interval:1	204	286	230.8	Above median	0.027	0.062
Lachnospira (117)	interval:1	207	357	242.1	Above median	0.002	0.021
Roseburia (133)	interval:1	257	381	150.6	Above median	0.002	0.021
Blautia (47)	interval:1	277	381	198.6	Above median	0.023	0.054
Moryella (71)	interval:1	317	381	85.6	Above median	0.065	0.092
Blautia (28)	interval:2	362	381	87.5	Above median	0.015	0.053

Supplementary Table 7. Differentially abundant OTUs of the Lachnospiraceae family in the first 12 months of life between infants with above vs. below median anti-MenC IgG levels
Differential abundance analysis was performed using two-sided smoothing spline ANOVA (ss-ANOVA). All analyses were corrected for time between vaccination and IgG measurements and for multiple comparisons.

	Anti-Ps6B IgG response			Anti-MenC IgG response		
	High	Low	*p*-value	High	Low	*p*-value
n	49	50		33	31	
Escherichia coli presence (%)	34 (69.4)	25 (50.0)	0.078	23 (69.7)	19 (61.3)	0.657
Klebsiella spp. presence (%)	23 (46.9)	21 (42.0)	0.770	16 (48.5)	13 (41.9)	0.783
Enterococcus spp. presence (%)	29 (59.2)	37 (74.0)	0.177	22 (66.7)	19 (61.3)	0.851

Supplementary Table 8. Validation of 16S rRNA gene sequencing-based results by targeted qPCR

Presence of *E. coli*, *Enterococcus* spp. and *Klebsiella* spp. identified by quantitative polymerase chain reaction (qPCR) on all week 1 samples, for infants with high and low anti-Ps6B and anti-MenC IgG responses. *p*-values were calculated with two-sided chi-square tests.
