# Supplementary material for: Mode of delivery modulates the intestinal microbiota and impacts the response to vaccination
Source: Nat Commun. 2022 Nov 15;13:6638. doi: 10.1038/s41467-022-34155-2 (PMC9666625; doi:10.1038/s41467-022-34155-2)
Supplement: Supplementary file 3 — Reporting Summary [file 41467_2022_34155_MOESM3_ESM.pdf]

nature portfolio

Corresponding author(s): Debby Bogaert
Last updated by author(s): Aug 29, 2022

Reporting Summary

Nature Portfolio wishes to improve the reproducibility of the work that we publish. This form provides structure for consistency and transparency in reporting. For further information on Nature Portfolio policies, see our Editorial Policies and the Editorial Policy Checklist.

Statistics

For all statistical analyses, confirm that the following items are present in the figure legend, table legend, main text, or Methods section.

n/a	Confirmed	
☐	☒	The exact sample size (n) for each experimental group/condition, given as a discrete number and unit of measurement
☐	☒	A statement on whether measurements were taken from distinct samples or whether the same sample was measured repeatedly
☐	☒	The statistical test(s) used AND whether they are one- or two-sided<br>*Only common tests should be described solely by name; describe more complex techniques in the Methods section.*
☐	☒	A description of all covariates tested
☐	☒	A description of any assumptions or corrections, such as tests of normality and adjustment for multiple comparisons
☐	☒	A full description of the statistical parameters including central tendency (e.g. means) or other basic estimates (e.g. regression coefficient) AND variation (e.g. standard deviation) or associated estimates of uncertainty (e.g. confidence intervals)
☐	☒	For null hypothesis testing, the test statistic (e.g. F, t, r) with confidence intervals, effect sizes, degrees of freedom and P value noted<br>*Give P values as exact values whenever suitable.*
☒	☐	For Bayesian analysis, information on the choice of priors and Markov chain Monte Carlo settings
☒	☐	For hierarchical and complex designs, identification of the appropriate level for tests and full reporting of outcomes
☐	☒	Estimates of effect sizes (e.g. Cohen's d, Pearson's r), indicating how they were calculated

Our web collection on statistics for biologists contains articles on many of the points above.

Software and code

Policy information about availability of computer code

Data collection	Glean Study Manager™ was used to build a database for data collection. This is based on FAB™ version 2.10.1, developed by Sidekick-IT.
Data analysis	Data analysis was performed in R version 4.0.3 within R studio version 1.3.1093. Primarily the R packages phyloseq (version 1.38.0), vegan (version 2.5-7), DirichletMultinomial (version 1.36.0), metagenomeSeq (version 1.36.0) and agricolae (version 1.3-5) were used in the analysis. All R code is publicly available on GitLab at: https://gitlab.com/EMdK/muis_vaccine_responses.

For manuscripts utilizing custom algorithms or software that are central to the research but not yet described in published literature, software must be made available to editors and reviewers. We strongly encourage code deposition in a community repository (e.g. GitHub). See the Nature Portfolio guidelines for submitting code & software for further information.

Data

Policy information about availability of data

All manuscripts must include a data availability statement. This statement should provide the following information, where applicable:

- Accession codes, unique identifiers, or web links for publicly available datasets
- A description of any restrictions on data availability
- For clinical datasets or third party data, please ensure that the statement adheres to our policy

Sequence data that support the findings of this study have been deposited in the NCBI Sequence Read Archive (SRA) database with BioProject ID PRJNA481243 [https://www.ncbi.nlm.nih.gov/bioproject/481243], and PRJNA555020 [https://www.ncbi.nlm.nih.gov/bioproject/555020]. The vaccine response data and relevant participant metadata are provided in the Source Data file. Additional participant metadata and data dictionaries can be made available after approval of a proposal. Taxonomic annotations were based on the Silva reference database (version 119).

March 2021

Field-specific reporting

Please select the one below that is the best fit for your research. If you are not sure, read the appropriate sections before making your selection.

- [x] Life sciences
- [ ] Behavioural & social sciences
- [ ] Ecological, evolutionary & environmental sciences

For a reference copy of the document with all sections, see nature.com/documents/nr-reporting-summary-flat.pdf

Life sciences study design

All studies must disclose on these points even when the disclosure is negative.

Sample size	The study had originally been powered based on the abundance and distribution of previously published microbiota data from infants, ensuring a power of 0.8 to detect at least significant differences in alpha and beta diversity between delivery mode groups as well as differences in abundance of the 25 most important operational taxonomical units (OTUs), taking into consideration OTUs with high and low variability and abundance and varying effect sizes. This power calculation was later verified by an online (HMP-based) tool. We initially aimed to enroll 88 infants, 44 infants per delivery mode group, allowing a drop-out of 10%. Due to uneven enrolment in both arms, approval was granted by the ethical committee to prolong enrolment to ensure sufficient caesarean section (CS) recruitment, simultaneously continuing the parallel enrolment of vaginally delivered (VD) infants to prevent seasonal/annual differences in microbiota development between the delivery mode groups. Eventually, 78 VD and 52 CS children were recruited; 10 (7.7%) dropped out after an average of 2 weeks of follow-up.
Data exclusions	Microbiome data were excluded from the analysis if fecal samples had insufficient bacterial DNA available (n=104). Antibody measurements were excluded from the analyses if infants did not receive their vaccinations in time (n=8 at month 12, n=1 at month 18), or if the saliva sample did not have a sufficient volume for laboratory analysis (n=8 at month 12, n=11 at month 18).
Replication	Confirmatory shotgun sequencing was performed on a subset of samples (n=20) to validate the major 16S-rRNA-based sequencing results. Furthermore, targeted species-specific quantitative PCR was performed for a panel of biomarker species on a large set of samples (n=119), to validate our 16S-rRNA-based sequencing results on species level. Biomarker species correlated well between the shotgun sequencing and the 16S-rRNA-based sequencing results, validating our findings obtained using 16S-rRNA-sequencing.
Randomization	Randomization was not applicable for this observational study.
Blinding	Blinding was not applicable for this observational study.

Reporting for specific materials, systems and methods

We require information from authors about some types of materials, experimental systems and methods used in many studies. Here, indicate whether each material, system or method listed is relevant to your study. If you are not sure if a list item applies to your research, read the appropriate section before selecting a response.

Materials & experimental systems

n/a	Involved in the study	
[ ]	[x]	Antibodies
[x]	[ ]	Eukaryotic cell lines
[x]	[ ]	Palaeontology and archaeology
[x]	[ ]	Animals and other organisms
[ ]	[x]	Human research participants
[ ]	[x]	Clinical data
[x]	[ ]	Dual use research of concern

Methods

n/a	Involved in the study	
[x]	[ ]	ChIP-seq
[x]	[ ]	Flow cytometry
[x]	[ ]	MRI-based neuroimaging

Antibodies

Antibodies used	Standard reference sera with previously assigned concentrations of serotype-specific IgG were an in-house intravenous immunoglobulin (IVIG) for pneumococcal serotypes (Sanquin, Amsterdam, The Netherlands), and CDC1992 for MenC (NIBSC, Ridge, United Kingdom).
Validation	Validation of these reference sera was previously performed, which has been described in the following publications:<br>- Elberse KEM, Tcherniaeva I, Berbers GAM, Schouls LM. Optimization and application of a multiplex bead-based assay to quantify serotype-specific IgG against Streptococcus pneumoniae polysaccharides: Response to the booster vaccine after immunization with the pneumococcal 7-valent conjugate vaccine. Clinical and Vaccine Immunology 2010; 17: 674–82.<br>- Holder PK, Maslanka SE, Pais LB, Dykes J, Plikaytis BD, Carlone GM. Assignment of Neisseria meningitidis serogroup A and C class-specific anticapsular antibody concentrations to the new standard reference serum CDC1992. Clinical and Diagnostic Laboratory Immunology 1995; 2: 132–7.

Human research participants

Policy information about studies involving human research participants

Population characteristics	The study population consists of healthy, term-born, Dutch infants. The relevant population characteristics are detailed in the baseline table (Table 1).
Recruitment	Microbiota, antibody and clinical data were available from 120 children, who participated in the prospective Microbiome Utrecht Infant Study (MUIS) consisting of healthy, full term Dutch children. Recruitment took place during pregnancy during regular pregnancy check-ups. All women giving birth vaginally or by caesarean section were eligible for the study. After delivery, all eligible infants were enrolled in the study unless interfering criteria hindered enrolment (severe perinatal/ neonatal/maternal complications as asphyxia, resuscitation, transfer to neonatal intensive care unit, etc; major congenital anomalies, language barrier, intention to move outside the research area; parents under the age of 18 years).<br>We aimed to recruit an open, healthy study population of infants, therefore - in addition to gynaecology departments - we specifically recruited parents at outpatient midwifery clinics. Regardless, we cannot rule out selection bias, as it is known that highly educated parents are more likely to participate in research.
Ethics oversight	Ethical approval was granted by the national ethics committee in the Netherlands, METC Noord-Holland (committee on research involving human subjects, M012-015). Both parents provided written informed consent.

Note that full information on the approval of the study protocol must also be provided in the manuscript.

Clinical data

Policy information about clinical studies

All manuscripts should comply with the ICMJEguidelines for publication of clinical research and a completedCONSORT checklist must be included with all submissions.

Clinical trial registration	The MUIS study is registered in the Netherlands Trial Register under number NTR3986.
Study protocol	The full study protocol is available upon request.
Data collection	Recruitment took place on the gynaecology and obstetrics departments of the Spaarneziekenhuis in Hoofddorp and the Kennemer Gasthuis in Haarlem and at midwifery clinics in regions Groot Kennemerland and Bollenstreek. All participants were born between December 2012 and November 2014. Samples and data were collected during home visits by trained research personnel.
Outcomes	The primary research aim of this work was to investigate associations between early-life exposures, gut microbiota development over the first year of life, and antibody responses to pneumococcal and meningococcal conjugate vaccination at the age of 12 and 18 months, respectively. Antibody concentrations were measured in saliva using multiplex immunoassays. Gut microbiota compositon was characterized using 16S rRNA gene sequencing at 10 time points in the first year of life. Detailed participant data was available from medical records and questionnaires obtained from parents at the same 10 time points and additionally at the age of 3 months. Secondary research objectives were not defined for the current work.
